# Malignant Ischemic Stroke in a Young Female: A Rare Primary Manifestation of Takayasu Arteritis

**DOI:** 10.1155/2019/7942825

**Published:** 2019-02-18

**Authors:** Bhupendra Shah, Roshan Chhetri

**Affiliations:** Department of Internal Medicine, B.P. Koirala Institute of Health Sciences, Dharan, Nepal

## Abstract

Takayasu arteritis is a rare chronic progressive granulomatous necrotizing large vessel panvasculitis mainly involving aorta and its main branches. It commonly affects the female in second to third decades. Common clinical features of Takayasu arteritis are hypertension, claudication, dizziness, headache, or fever. Takayasu arteritis is diagnosed with clinical history of claudication, absent pulse, discordant blood pressure, bruit over aorta, and typical angiographic findings. Stroke as a primary manifestation of Takayasu arteritis is rarely reported in the medical literatures. We are reporting a 16-year-old female who had malignant ischemic stroke as a first manifestation of Takayasu arteritis.

## 1. Introduction

Takayasu arteritis, named after Japanese professor of Ophthalmology Mikito Takayasu [1], is a chronic progressive large vessel necrotizing granulomatous panvasculitis mainly involving aorta and its main branches [2]. It commonly affects the female of second to third decade. Mwipatayi et al. reported that the common clinical features of Takayasu arteritis were hypertension, cardiac failure, deranged renal function, cerebrovascular diseases, claudication, or gangrene [3]. Of neurological manifestations, dizziness, visual disturbances, headache, seizure, or ischemic stroke are common manifestations [4]. Hwang J et al. reported that the prevalence of stroke among patient with Takayasu arteritis was 11.1% [5]. However, stroke as a primary manifestation of Takayasu arteritis is a rare event. Hence, we report a case of 16-year-old girl who had malignant left hemispheric ischemic stroke as a first manifestation of Takayasu arteritis.

## 2. Case Reports

**Figure 1:**
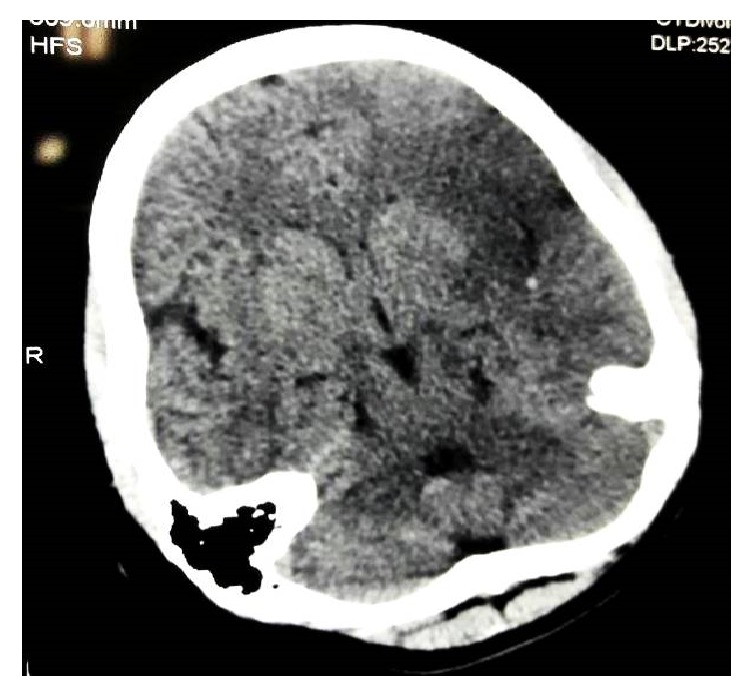
Computed tomography of head showed hypodensity at left frontotemporoparietal region along with middle cerebral artery dot sign.

**Figure 2:**
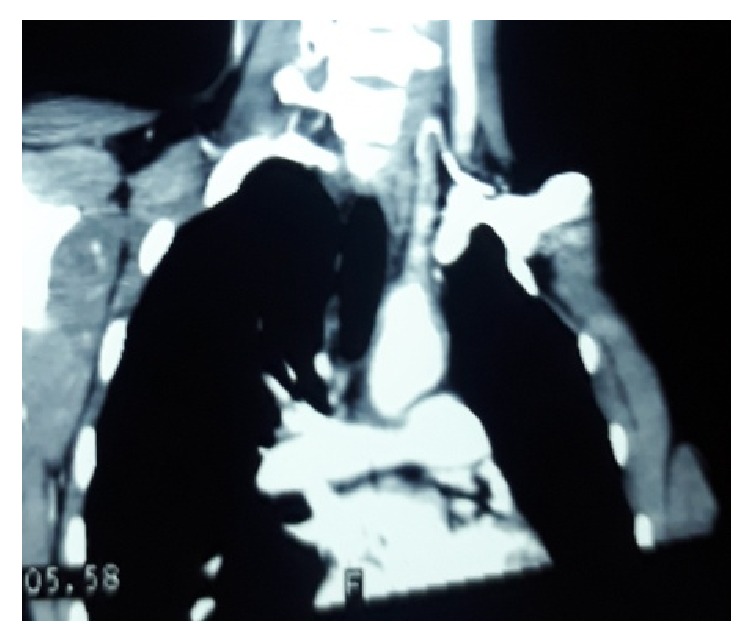
Computed tomographic aortogram revealed circumferential thickening of arch of aorta, brachiocephalic trunk, and right common carotid.

A 16-year-old girl presented to emergency room with complaints of weakness of right upper limb and lower limb for an 18-hour duration. She also had facial deviation towards left side. She had inability to produce and comprehend the speech. There was no history of fever, head trauma, and seizure. She had no significant past medical history and family history. She was nonsmoker and never consumed alcohol. At emergency room, her Glasgow coma scale score (GCS) was 11/15(E4V1M6). Her blood pressure was 110/80 mmHg, pulse rate 80 beat per minute in left radial artery and absent pulse in right brachial and radial artery, respiratory rate -16 cycle per minute, temperature -98°F. Central nervous system examination revealed right sided hemiplegia, global aphasia, and upper motor neuron type facial nerve paralysis. Other central nervous examination and systematic examination findings were normal. The baseline investigation reports were illustrated in Table 1. Computed tomography of head showed hypodensity at left frontotemporoparietal region along with middle cerebral artery dot sign (Figure 1). Patient was admitted with provisional diagnosis of ischemic malignant lobar stroke with modified ranking scale of 5/6. Ultrasonography Doppler revealed thickening of right common carotid, right internal carotid, right external carotid, and right subclavian artery with significant lumen obstruction. Computed tomographic aortogram revealed circumferential thickening of arch of aorta, brachiocephalic trunk, and right common carotid (Figure 2). Electrocardiogram, Chest X-RAY, Ultrasound abdomen and Echocardiography was normal. We made final diagnoses of malignant ischemic stroke secondary to Takayasu arteritis. She was treated with tablet aspirin 75 mg once daily, tab, and prednisolone 40 mg once daily. Physiotherapy was initiated on second day of admission. She was discharged from hospital on tenth day of admission on prednisolone and aspirin. At the time of discharge her stroke disability modified ranking scale was 3/5.

## 3. Discussion

We report a case of 16-year-old girl who had left hemispheric ischemic stroke. She had absent pulse in the right brachial artery, discordant blood pressure in two arms, and aortogram abnormality; hence we diagnose her as a case of Takayasu arteritis presenting as ischemic stroke based on American Rheumatology Criteria as illustrated in the Table 2 [6].

Our patient had ischemic stroke as a first presentation of the Takayasu arteritis. Similar case report of ischemic stroke in a patient with Takayasu arteritis was done by Gao S et al. from China [7] and Silver M et al. [8] while Hwang J et al. reported that the most common form of stroke in Takayasu arteritis was large lobar [5] stroke similar to our case. Most of the published literatures showed that stroke can occur as one of multiple manifestations of the Takayasu arteritis; however, the stroke as sole and primary presentation of Takayasu arteritis is rarely reported in the medical literatures; hence, the malignant ischemic stroke in our patient was a rare manifestation of Takayasu arteritis. Despite hypertension, traditional risk factor of stroke is reported in up to 77% in a patient with Takayasu arteritis as by Mwipatayi BP et al. [3] our patient had normal blood pressure. The other risk factors of stroke were absent in our case.

Our patient had new onset of stroke, decreased pulse volume in brachial artery, and elevated ESR and CRP which suggest the active form of Takayasu arteries as reported by the Direskeneli H et al. [9]. Kesar G et al. in their systematic review on management of Takayasu arteritis reported that corticosteroids and immunosuppressive like methotrexate, cyclophosphamide, azathioprine, and leflunomide are commonly prescribed medication for active Takayasu arteritis [10]. Ito I reported that the corticosteroids alone were effective in 20 to 75% patient with active Takayasu arteritis [11]. Hence, we managed our patient with Takayasu arteritis with malignant ischemic stroke with prednisolone and aspirin. This case report highlights the significance of measuring pulse volume and blood pressure in two arms in a young patient with ischemic stroke which may lead to suspicion of Takayasu arteritis. Diagnosing the Takayasu arteritis and treating it with immunosuppressive might differ in the outcome of the patient.

## Figures and Tables

**Table 1:** Baseline laboratory reports of the patient.

Laboratory parameters	Values	Normal range

Haemoglobin	9.5 gm/dl	11-16 gm/dl
Total leucocyte count	14300	4000-11000 cell/mm3
Platelet count	430000 cell/mm3	150000-400000 cell/mm3
Glucose	122 mg/dl	<140 mg/dl
Urea	11 mg/dl	10-50 mg/dl
Creatine	0.2	0.5-1.4 mg/dl
Sodium	138 mmol/L	136-145 mmol/L
Potassium	4.4 mmol/l	3.5-5 mmol/l
ANA	5.68 U/ml	<10.00 IU/ml
Anti ds DNA	0.24	<0.9 IU/ml

*Abbreviations.* ANA-antinuclear antibody, Anti ds DNA: anti-double stranded deoxyribonucleic acid.

**Table 2:** American Rheumatology Criteria for diagnosing Takayasu arteritis.

1.	Age at disease onset ≤40 years
2.	Claudication of extremities
3.	Decreased arterial pulse
4.	Blood pressure difference >10 mmHg
5.	Bruits over subclavian or aorta
6.	Arteriogram abnormality

To diagnose the Takayasu arteritis patient should fulfill at least 3 criteria. Our patient had fulfilled criteria 1, 3, 4, 5, and 6.
